# 2,3,4-Tri-*O*-acetyl-β-d-xylosyl 2,4-dichloro­phenoxy­acetate

**DOI:** 10.1107/S1600536808005837

**Published:** 2008-03-05

**Authors:** Xiaoming Wang, Xinyuan Li, Yale Yin, Yanjun Pang, Yonghua Yang

**Affiliations:** a State Key Laboratory of Pharmaceutical Biotechnology, School of Life Sciences, nanjing University, Hankou Road, Nanjing 210093, People’s Republic of China

## Abstract

In the title compound, C_19_H_20_Cl_2_O_10_, the hexopyranosyl ring adopts a chair conformation. The four substituents are in equatorial positions. The mol­ecules arelinked *via* C—H⋯O contacts along the *a* axis.

## Related literature

For related literature, see: Hamner *et al.* (1946 ▶); Chandra­sekhar & Pattabhi (1977 ▶); Dalton (2004 ▶); Tsorteki *et al.* (2004 ▶); Yang *et al.* (2004 ▶).
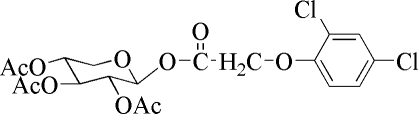


## Experimental

### 

#### Crystal data

C_19_H_20_Cl_2_O_10_
*M* *_r_* = 479.25
Monoclinic, 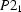

*a* = 5.6601 (8) Å
*b* = 23.129 (3) Å
*c* = 8.7456 (13) Å
β = 104.281 (2)°
*V* = 1109.5 (3) Å^3^
*Z* = 2
Mo *K*α radiation
μ = 0.34 mm^−1^
*T* = 293 (2) K
0.45 × 0.23 × 0.21 mm

#### Data collection

Bruker SMART APEX CCD diffractometer
Absorption correction: none
5656 measured reflections
3337 independent reflections
3044 reflections with *I* > 2σ(*I*)
*R*_int_ = 0.089

#### Refinement

*R*[*F* ^2^ > 2σ(*F* ^2^)] = 0.046
*wR*(*F* ^2^) = 0.121
*S* = 1.03
3337 reflections
284 parameters
1 restraint
H-atom parameters constrained
Δρ_max_ = 0.29 e Å^−3^
Δρ_min_ = −0.31 e Å^−3^
Absolute structure: Flack (1983 ▶), 1325 Friedel pairs
Flack parameter: 0.04 (8)

### 

Data collection: *SMART* (Bruker, 2003 ▶); cell refinement: *SAINT-Plus* (Bruker, 2003 ▶); data reduction: *SAINT-Plus*; program(s) used to solve structure: *SHELXS97* (Sheldrick, 2008 ▶); program(s) used to refine structure: *SHELXL97* (Sheldrick, 2008 ▶); molecular graphics: *SHELXTL* (Sheldrick, 2008 ▶); software used to prepare material for publication: *SHELXTL*.

## Supplementary Material

Crystal structure: contains datablocks global, I. DOI: 10.1107/S1600536808005837/si2071sup1.cif

Structure factors: contains datablocks I. DOI: 10.1107/S1600536808005837/si2071Isup2.hkl

Additional supplementary materials:  crystallographic information; 3D view; checkCIF report

## Figures and Tables

**Table 1:** Hydrogen-bond geometry (Å, °)

*D*—H⋯*A*	*D*—H	H⋯*A*	*D*⋯*A*	*D*—H⋯*A*
C3—H3⋯O6^i^	0.93	2.41	3.322 (6)	168
C9—H9⋯O10^i^	0.98	2.54	3.381 (4)	144
C11—H11⋯O10^i^	0.98	2.44	3.296 (4)	146

Symmetry code: (i) 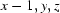.

**Fig. 1.**
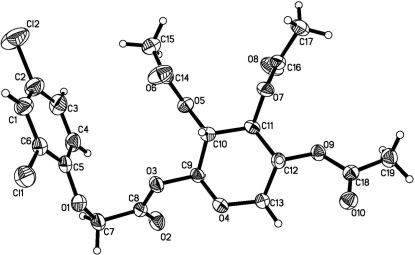
The molecular structure of the title compound, showing the atom-labelling scheme. Displacement ellipsoids are drawn at the 50% probability level.

## supplementary crystallographic information

### Comment

The plant growth regulator 2,4-dichlorophenoxyacetic acid cocrystallized as a guest molecule in Heptakis(2,3,6-tri-*O*-methyl)-β-cyclodextrin (Tsorteki *et al.*, 2004), and it plays an important role in graining and controlling weeds (Hamner *et al.*, 1946). However, problems such as toxic residues and environmental pollution were protruded increasingly by using amounts of herbicides during the past decades (Dalton, 2004). In order to search for a new herbicide with high efficiency and low toxicity, we obtained the title compound. All bond lengths and angles in the title molecule show normal values. The hexopyranosyl ring adopts a chair conformation (Fig. 1). The three acetyl groups are individually planar and occupy equatorial positions (Yang *et al.*, 2004). The 2,4-dichlorophenoxyacetic acid group shows a similar geometry in 2-Chlorophenoxyacetic acid (Chandrasekhar & Pattabhi, 1977), and it is twisted at the bond of O3—C8—C7—O1, with the torsion angle of 4.3°. The title molecules are linked *via* intermolecular hydrogen bonding C—H···O contacts along the *a* axis by translation (Table 1).

### Experimental

The title compound was prepared from α-*D*-1-bromo-2,3,4-tri-*O*- acetyl-xylosyl with 2,4-dichlorophenoxyacetic acid in aq NaOH at the benzyltriethylammonium chloride and 4-dimethylaminopyridine in present. Fine block colourless crystals for single-crystal X-ray diffraction were obtained by slow evaporation of an ethyl acetate at room temperature.

### Refinement

The H atoms were refined by riding on their appropriate parent atoms in their as-found or calculated positions. The C—H distances for CH, CH_2_ and CH_3_ groups are 0.93, 0.96 and 0.97 Å, respectively, with *U*_iso_(H) = 1.2*U*_eq_(C*sp*^2^) or 1.5*U*_eq_(C*sp*^3^). The absolute structure parameter was determined as 0.04 (8) (Flack, 1983). The number of Friedel pairs was found to be 1325 by comparison of merged intensity reflections (2012) with unmerged unique reflections of the final refinement.

### Crystal data

**Table:**

C_19_H_20_Cl_2_O_10_	*F*_000_ = 496
*M**_r_* = 479.25	*D*_x_ = 1.435 Mg m^−^^3^
Monoclinic, *P*2_1_	Mo *K*α radiation λ = 0.71073 Å
Hall symbol: P 2yb	Cell parameters from 90 reflections
*a* = 5.6601 (8) Å	θ = 2.4–25.0º
*b* = 23.129 (3) Å	µ = 0.35 mm^−^^1^
*c* = 8.7456 (13) Å	*T* = 293 (2) K
β = 104.281 (2)º	Block, colourless
*V* = 1109.5 (3) Å^3^	0.45 × 0.23 × 0.21 mm
*Z* = 2	

### Data collection

**Table:**

Bruker SMART APEX CCD diffractometer	3337 independent reflections
Radiation source: fine-focus sealed tube	3044 reflections with *I* > 2σ(*I*)
Monochromator: graphite	*R*_int_ = 0.089
Detector resolution: 9.00cm pixels mm^-1^	θ_max_ = 25.0º
*T* = 293(2) K	θ_min_ = 2.4º
ω and φ scans	*h* = −6→5
Absorption correction: none	*k* = −27→15
5656 measured reflections	*l* = −9→10

### Refinement

**Table:**

Refinement on *F*^2^	Hydrogen site location: inferred from neighbouring sites
Least-squares matrix: full	H-atom parameters constrained
*R*[*F*^2^ > 2σ(*F*^2^)] = 0.046	*w* = 1/[σ^2^(*F*_o_^2^) + (0.0733*P*)^2^ + 0.2029*P*] where *P* = (*F*_o_^2^ + 2*F*_c_^2^)/3
*wR*(*F*^2^) = 0.121	(Δ/σ)_max_ < 0.001
*S* = 1.04	Δρ_max_ = 0.29 e Å^−^^3^
3337 reflections	Δρ_min_ = −0.31 e Å^−^^3^
284 parameters	Extinction correction: none
1 restraint	Absolute structure: Flack (1983), 1325 Friedel pairs
Primary atom site location: structure-invariant direct methods	Flack parameter: 0.04 (8)
Secondary atom site location: difference Fourier map	

### Special details

**Table:**

Geometry. All e.s.d.'s (except the e.s.d. in the dihedral angle between two l.s. planes) are estimated using the full covariance matrix. The cell e.s.d.'s are taken into account individually in the estimation of e.s.d.'s in distances, angles and torsion angles; correlations between e.s.d.'s in cell parameters are only used when they are defined by crystal symmetry. An approximate (isotropic) treatment of cell e.s.d.'s is used for estimating e.s.d.'s involving l.s. planes.
Refinement. Refinement of *F*^2^ against ALL reflections. The weighted *R*-factor *wR* and goodness of fit *S* are based on *F*^2^, conventional *R*-factors *R* are based on *F*, with *F* set to zero for negative *F*^2^. The threshold expression of *F*^2^ > σ(*F*^2^) is used only for calculating *R*-factors(gt) *etc*. and is not relevant to the choice of reflections for refinement. *R*-factors based on *F*^2^ are statistically about twice as large as those based on *F*, and *R*- factors based on ALL data will be even larger.

### Fractional atomic coordinates and isotropic or equivalent isotropic displacement parameters (Å^2^)

**Table:**

	*x*	*y*	*z*	*U*_iso_*/*U*_eq_	
Cl1	0.39611 (18)	0.41178 (5)	0.40852 (13)	0.0708 (3)	
Cl2	−0.4306 (3)	0.37317 (8)	−0.0324 (2)	0.1303 (7)	
O1	0.2663 (5)	0.31057 (11)	0.5567 (3)	0.0593 (6)	
O2	0.1992 (6)	0.16524 (12)	0.6787 (3)	0.0662 (7)	
O3	0.3421 (4)	0.20235 (10)	0.4807 (3)	0.0492 (5)	
O4	0.6349 (4)	0.13402 (10)	0.5269 (3)	0.0501 (6)	
O5	0.1431 (4)	0.15293 (10)	0.1744 (3)	0.0452 (5)	
O6	0.2612 (5)	0.22405 (14)	0.0364 (4)	0.0748 (9)	
O7	0.4821 (4)	0.08031 (10)	0.0669 (2)	0.0478 (5)	
O8	0.1649 (6)	0.01975 (16)	0.0038 (4)	0.0854 (10)	
O9	0.7485 (4)	0.00745 (10)	0.2985 (2)	0.0451 (5)	
O10	1.1379 (4)	0.01306 (11)	0.4309 (3)	0.0552 (6)	
C1	−0.0205 (8)	0.38597 (19)	0.1966 (5)	0.0648 (10)	
H1	0.0105	0.4181	0.1406	0.078*	
C2	−0.2255 (8)	0.3534 (2)	0.1430 (5)	0.0733 (11)	
C3	−0.2759 (8)	0.30616 (19)	0.2235 (6)	0.0724 (11)	
H3	−0.4165	0.2846	0.1841	0.087*	
C4	−0.1159 (7)	0.29073 (17)	0.3640 (5)	0.0601 (9)	
H4	−0.1510	0.2592	0.4207	0.072*	
C5	0.0968 (6)	0.32194 (15)	0.4211 (4)	0.0502 (8)	
C6	0.1387 (6)	0.36984 (16)	0.3357 (4)	0.0520 (8)	
C7	0.2153 (9)	0.26725 (17)	0.6570 (4)	0.0632 (10)	
H7A	0.0481	0.2720	0.6641	0.076*	
H7B	0.3196	0.2734	0.7617	0.076*	
C8	0.2479 (6)	0.20569 (15)	0.6091 (4)	0.0470 (7)	
C9	0.3960 (5)	0.14523 (14)	0.4394 (3)	0.0406 (6)	
H9	0.2811	0.1173	0.4647	0.049*	
C10	0.3903 (5)	0.14345 (14)	0.2641 (3)	0.0396 (6)	
H10	0.5002	0.1726	0.2382	0.048*	
C11	0.4660 (5)	0.08292 (14)	0.2273 (3)	0.0383 (6)	
H11	0.3442	0.0550	0.2435	0.046*	
C12	0.7106 (5)	0.06757 (13)	0.3323 (4)	0.0407 (6)	
H12	0.8380	0.0917	0.3066	0.049*	
C13	0.7069 (6)	0.07587 (15)	0.5044 (4)	0.0462 (7)	
H13A	0.5931	0.0489	0.5323	0.055*	
H13B	0.8676	0.0684	0.5719	0.055*	
C14	0.1046 (6)	0.19246 (16)	0.0576 (4)	0.0479 (8)	
C15	−0.1479 (7)	0.1900 (2)	−0.0418 (5)	0.0628 (10)	
H15A	−0.2249	0.2269	−0.0404	0.094*	
H15B	−0.2378	0.1610	−0.0013	0.094*	
H15C	−0.1450	0.1804	−0.1481	0.094*	
C16	0.3333 (7)	0.04317 (17)	−0.0295 (4)	0.0543 (9)	
C17	0.4131 (10)	0.0352 (2)	−0.1786 (5)	0.0802 (14)	
H17A	0.5523	0.0101	−0.1593	0.120*	
H17B	0.4557	0.0721	−0.2148	0.120*	
H17C	0.2827	0.0184	−0.2575	0.120*	
C18	0.9736 (5)	−0.01395 (15)	0.3484 (4)	0.0439 (7)	
C19	0.9926 (7)	−0.07338 (17)	0.2889 (5)	0.0620 (9)	
H19A	1.0063	−0.0716	0.1818	0.093*	
H19B	0.8497	−0.0950	0.2934	0.093*	
H19C	1.1343	−0.0920	0.3531	0.093*	

### Atomic displacement parameters (Å^2^)

**Table:**

	*U*^11^	*U*^22^	*U*^33^	*U*^12^	*U*^13^	*U*^23^
Cl1	0.0728 (6)	0.0576 (6)	0.0782 (6)	−0.0171 (5)	0.0114 (5)	−0.0004 (5)
Cl2	0.1225 (12)	0.1201 (14)	0.1079 (10)	−0.0217 (10)	−0.0481 (9)	0.0335 (10)
O1	0.0780 (16)	0.0361 (13)	0.0569 (14)	0.0026 (12)	0.0038 (12)	−0.0004 (11)
O2	0.100 (2)	0.0457 (16)	0.0624 (16)	0.0022 (14)	0.0379 (14)	0.0033 (12)
O3	0.0682 (14)	0.0353 (13)	0.0468 (12)	0.0000 (10)	0.0191 (10)	−0.0025 (10)
O4	0.0557 (13)	0.0461 (15)	0.0444 (12)	0.0009 (10)	0.0044 (10)	−0.0115 (10)
O5	0.0425 (11)	0.0481 (14)	0.0462 (12)	−0.0016 (9)	0.0130 (9)	0.0082 (10)
O6	0.0692 (16)	0.076 (2)	0.0702 (17)	−0.0201 (15)	−0.0009 (13)	0.0305 (15)
O7	0.0600 (13)	0.0497 (14)	0.0362 (10)	−0.0045 (11)	0.0170 (9)	−0.0007 (10)
O8	0.090 (2)	0.091 (3)	0.0718 (19)	−0.0339 (19)	0.0142 (16)	−0.0312 (17)
O9	0.0458 (11)	0.0385 (13)	0.0508 (12)	−0.0028 (9)	0.0114 (9)	−0.0053 (10)
O10	0.0480 (12)	0.0565 (16)	0.0585 (14)	−0.0034 (11)	0.0084 (10)	−0.0008 (12)
C1	0.075 (2)	0.057 (2)	0.058 (2)	−0.0028 (19)	0.0095 (17)	0.0090 (18)
C2	0.070 (2)	0.067 (3)	0.071 (2)	0.001 (2)	−0.0058 (19)	0.006 (2)
C3	0.065 (2)	0.050 (2)	0.095 (3)	−0.0128 (18)	0.003 (2)	−0.003 (2)
C4	0.068 (2)	0.037 (2)	0.074 (2)	−0.0035 (16)	0.0142 (18)	0.0021 (17)
C5	0.0619 (19)	0.0340 (18)	0.0543 (18)	0.0064 (15)	0.0134 (15)	−0.0033 (14)
C6	0.0598 (18)	0.0414 (19)	0.0548 (18)	0.0017 (15)	0.0142 (15)	−0.0048 (15)
C7	0.097 (3)	0.041 (2)	0.052 (2)	0.0103 (19)	0.0190 (19)	−0.0014 (17)
C8	0.0599 (18)	0.0381 (19)	0.0414 (15)	0.0056 (14)	0.0095 (14)	0.0016 (14)
C9	0.0506 (16)	0.0316 (16)	0.0395 (15)	−0.0021 (13)	0.0112 (12)	−0.0031 (12)
C10	0.0399 (14)	0.0401 (17)	0.0389 (14)	−0.0072 (12)	0.0099 (11)	0.0027 (13)
C11	0.0443 (14)	0.0404 (17)	0.0331 (13)	−0.0098 (12)	0.0150 (11)	−0.0017 (12)
C12	0.0418 (15)	0.0334 (17)	0.0477 (16)	−0.0036 (13)	0.0125 (12)	−0.0020 (13)
C13	0.0502 (16)	0.0423 (19)	0.0442 (16)	0.0044 (14)	0.0081 (13)	−0.0024 (14)
C14	0.0517 (17)	0.051 (2)	0.0423 (16)	−0.0055 (15)	0.0146 (13)	−0.0017 (14)
C15	0.0564 (19)	0.073 (3)	0.058 (2)	0.0036 (18)	0.0110 (16)	0.0126 (19)
C16	0.069 (2)	0.046 (2)	0.0420 (17)	0.0046 (17)	0.0039 (16)	−0.0052 (15)
C17	0.132 (4)	0.065 (3)	0.044 (2)	0.019 (3)	0.023 (2)	−0.0076 (19)
C18	0.0437 (17)	0.0473 (19)	0.0421 (16)	−0.0012 (14)	0.0136 (13)	0.0055 (13)
C19	0.063 (2)	0.051 (2)	0.071 (2)	0.0030 (17)	0.0147 (17)	−0.0049 (18)

### Geometric parameters (Å, °)

**Table:**

Cl1—C6	1.735 (4)	C5—C6	1.389 (5)
Cl2—C2	1.740 (4)	C7—C8	1.508 (5)
O1—C5	1.355 (4)	C7—H7A	0.9700
O1—C7	1.407 (5)	C7—H7B	0.9700
O2—C8	1.185 (4)	C9—C10	1.526 (4)
O3—C8	1.359 (4)	C9—H9	0.9800
O3—C9	1.422 (4)	C10—C11	1.521 (4)
O4—C9	1.404 (4)	C10—H10	0.9800
O4—C13	1.433 (4)	C11—C12	1.503 (4)
O5—C14	1.347 (4)	C11—H11	0.9800
O5—C10	1.442 (4)	C12—C13	1.523 (4)
O6—C14	1.198 (4)	C12—H12	0.9800
O7—C16	1.344 (4)	C13—H13A	0.9700
O7—C11	1.429 (3)	C13—H13B	0.9700
O8—C16	1.193 (5)	C14—C15	1.479 (5)
O9—C18	1.336 (4)	C15—H15A	0.9600
O9—C12	1.448 (4)	C15—H15B	0.9600
O10—C18	1.202 (4)	C15—H15C	0.9600
C1—C2	1.366 (6)	C16—C17	1.493 (5)
C1—C6	1.375 (5)	C17—H17A	0.9600
C1—H1	0.9300	C17—H17B	0.9600
C2—C3	1.367 (6)	C17—H17C	0.9600
C3—C4	1.381 (6)	C18—C19	1.483 (5)
C3—H3	0.9300	C19—H19A	0.9600
C4—C5	1.387 (5)	C19—H19B	0.9600
C4—H4	0.9300	C19—H19C	0.9600
			
C5—O1—C7	118.3 (3)	O7—C11—C12	108.5 (2)
C8—O3—C9	114.5 (2)	O7—C11—C10	109.6 (2)
C9—O4—C13	111.5 (2)	C12—C11—C10	110.7 (2)
C14—O5—C10	118.0 (2)	O7—C11—H11	109.3
C16—O7—C11	117.4 (3)	C12—C11—H11	109.3
C18—O9—C12	117.8 (2)	C10—C11—H11	109.3
C2—C1—C6	118.0 (4)	O9—C12—C11	105.2 (2)
C2—C1—H1	121.0	O9—C12—C13	111.2 (2)
C6—C1—H1	121.0	C11—C12—C13	109.8 (2)
C1—C2—C3	122.1 (4)	O9—C12—H12	110.2
C1—C2—Cl2	118.9 (4)	C11—C12—H12	110.2
C3—C2—Cl2	119.1 (3)	C13—C12—H12	110.2
C2—C3—C4	119.4 (4)	O4—C13—C12	109.1 (3)
C2—C3—H3	120.3	O4—C13—H13A	109.9
C4—C3—H3	120.3	C12—C13—H13A	109.9
C3—C4—C5	120.4 (4)	O4—C13—H13B	109.9
C3—C4—H4	119.8	C12—C13—H13B	109.9
C5—C4—H4	119.8	H13A—C13—H13B	108.3
O1—C5—C4	125.3 (3)	O6—C14—O5	122.9 (3)
O1—C5—C6	116.6 (3)	O6—C14—C15	125.2 (3)
C4—C5—C6	118.0 (3)	O5—C14—C15	111.8 (3)
C1—C6—C5	122.1 (3)	C14—C15—H15A	109.5
C1—C6—Cl1	118.7 (3)	C14—C15—H15B	109.5
C5—C6—Cl1	119.2 (3)	H15A—C15—H15B	109.5
O1—C7—C8	116.2 (3)	C14—C15—H15C	109.5
O1—C7—H7A	108.2	H15A—C15—H15C	109.5
C8—C7—H7A	108.2	H15B—C15—H15C	109.5
O1—C7—H7B	108.2	O8—C16—O7	123.5 (3)
C8—C7—H7B	108.2	O8—C16—C17	126.1 (4)
H7A—C7—H7B	107.4	O7—C16—C17	110.4 (4)
O2—C8—O3	124.6 (3)	C16—C17—H17A	109.5
O2—C8—C7	122.9 (3)	C16—C17—H17B	109.5
O3—C8—C7	112.5 (3)	H17A—C17—H17B	109.5
O4—C9—O3	105.7 (2)	C16—C17—H17C	109.5
O4—C9—C10	108.8 (2)	H17A—C17—H17C	109.5
O3—C9—C10	109.1 (2)	H17B—C17—H17C	109.5
O4—C9—H9	111.0	O10—C18—O9	122.5 (3)
O3—C9—H9	111.0	O10—C18—C19	125.5 (3)
C10—C9—H9	111.0	O9—C18—C19	112.0 (3)
O5—C10—C11	108.1 (2)	C18—C19—H19A	109.5
O5—C10—C9	108.6 (2)	C18—C19—H19B	109.5
C11—C10—C9	107.4 (2)	H19A—C19—H19B	109.5
O5—C10—H10	110.8	C18—C19—H19C	109.5
C11—C10—H10	110.8	H19A—C19—H19C	109.5
C9—C10—H10	110.8	H19B—C19—H19C	109.5
			
C6—C1—C2—C3	0.4 (7)	O4—C9—C10—O5	178.0 (2)
C6—C1—C2—Cl2	179.7 (3)	O3—C9—C10—O5	−67.1 (3)
C1—C2—C3—C4	0.3 (7)	O4—C9—C10—C11	61.2 (3)
Cl2—C2—C3—C4	−179.0 (4)	O3—C9—C10—C11	176.1 (2)
C2—C3—C4—C5	−1.6 (7)	C16—O7—C11—C12	120.4 (3)
C7—O1—C5—C4	−6.4 (5)	C16—O7—C11—C10	−118.6 (3)
C7—O1—C5—C6	171.7 (3)	O5—C10—C11—O7	67.4 (3)
C3—C4—C5—O1	−179.8 (4)	C9—C10—C11—O7	−175.5 (2)
C3—C4—C5—C6	2.1 (5)	O5—C10—C11—C12	−172.9 (2)
C2—C1—C6—C5	0.2 (6)	C9—C10—C11—C12	−55.9 (3)
C2—C1—C6—Cl1	−178.3 (3)	C18—O9—C12—C11	165.2 (2)
O1—C5—C6—C1	−179.7 (3)	C18—O9—C12—C13	−76.0 (3)
C4—C5—C6—C1	−1.4 (5)	O7—C11—C12—O9	−66.0 (3)
O1—C5—C6—Cl1	−1.2 (4)	C10—C11—C12—O9	173.7 (2)
C4—C5—C6—Cl1	177.0 (3)	O7—C11—C12—C13	174.3 (2)
C5—O1—C7—C8	78.3 (4)	C10—C11—C12—C13	54.0 (3)
C9—O3—C8—O2	−4.1 (5)	C9—O4—C13—C12	63.7 (3)
C9—O3—C8—C7	174.8 (3)	O9—C12—C13—O4	−171.9 (2)
O1—C7—C8—O2	−176.0 (4)	C11—C12—C13—O4	−55.8 (3)
O1—C7—C8—O3	5.0 (5)	C10—O5—C14—O6	−8.1 (5)
C13—O4—C9—O3	176.0 (2)	C10—O5—C14—C15	169.6 (3)
C13—O4—C9—C10	−66.9 (3)	C11—O7—C16—O8	12.6 (5)
C8—O3—C9—O4	−87.9 (3)	C11—O7—C16—C17	−166.3 (3)
C8—O3—C9—C10	155.2 (3)	C12—O9—C18—O10	6.7 (4)
C14—O5—C10—C11	−112.4 (3)	C12—O9—C18—C19	−172.9 (3)
C14—O5—C10—C9	131.3 (3)		

### Hydrogen-bond geometry (Å, °)

**Table:**

*D*—H···*A*	*D*—H	H···*A*	*D*···*A*	*D*—H···*A*
C3—H3···O6^i^	0.93	2.41	3.322 (6)	168
C9—H9···O10^i^	0.98	2.54	3.381 (4)	144
C11—H11···O10^i^	0.98	2.44	3.296 (4)	146

Symmetry codes: (i) *x*−1, *y*, *z*.
